# Induction of the immunoprotective coat of *Yersinia pestis* at body temperature is mediated by the Caf1R transcription factor

**DOI:** 10.1186/s12866-019-1444-4

**Published:** 2019-03-29

**Authors:** Abdulmajeed D. Al-Jawdah, Iglika G. Ivanova, Helen Waller, Neil D. Perkins, Jeremy H. Lakey, Daniel T. Peters

**Affiliations:** Institute for Cell and Molecular Biosciences, Medical School, Newcastle University, Newcastle upon Tyne, UK

**Keywords:** Gene expression regulation, *Yersinia pestis*, AraC transcription factor, Temperature, Fimbriae, bacterial

## Abstract

**Background:**

Thermal regulation of gene expression occurs in many microorganisms, and is mediated via several typical mechanisms. *Yersinia pestis* is the causative agent of the plague and spreads by zoonotic transfer from fleas to mammalian blood with a concomitant rapid temperature change, from ambient to 37 °C, which induces the expression of capsular antigen (Caf1) that inhibits phagocytosis. Caf1 is formed into long polymeric fimbriae by a periplasmic chaperone (Caf1M) and outer membrane usher (Caf1A). All three are encoded on an operon regulated by an AraC-type transcription factor Caf1R. The aim of this study was to determine the role of Caf1R in the thermal control of *caf1* operon gene expression.

**Results:**

PCR analysis of cDNA demonstrated that the genes of the operon are transcribed as a single polycistronic mRNA. Bioinformatic analysis, supported by deletion mutagenesis, then revealed a region containing the promoter of this polycistronic transcript that was critical for Caf1 protein expression. Caf1R was found to be essential for Caf1 protein production. Finally, RT-PCR analysis and western blot experiments showed large, Caf1R dependent increases in *caf1* operon transcripts upon a shift in temperature from 25 °C to 35 °C.

**Conclusions:**

The results show that thermal control of Caf1 polymer production is established at the transcriptional level, in a Caf1R dependent manner. This gives us new insights into how a virulent pathogen evades destruction by the immune system by detecting and responding to environmental changes.

**Electronic supplementary material:**

The online version of this article (10.1186/s12866-019-1444-4) contains supplementary material, which is available to authorized users.

## Background

Temperature is an important stimulus for pathogens infecting mammalian and avian hosts, allowing them to differentiate between host and non-host environments. Microorganisms can employ a suite of different mechanisms to sense and respond to changes in temperature by altering their patterns of gene expression. These mechanisms can act at either the transcriptional or translational levels and can be mediated by protein, RNA or DNA [1]. Typical mechanisms include changes in the degree of supercoiling in DNA, as well as elements of local structure such as promoter curvature, which can affect transcription efficiency, when temperature increases or falls [1]. Another common mechanism involves RNA “thermometers” that form temperature dependent mRNA secondary structures that can inhibit translation below a critical temperature [1, 2]. In addition, some proteins can undergo conformational changes in response to alterations in temperature, resulting in the post-translational modification of downstream transcription factors [3, 4], inhibition of DNA binding [5, 6], or protein degradation [7], with subsequent changes in gene expression.

The bacterium *Yersinia pestis* is the etiologic agent of the plague, responsible for approximately 200 million deaths across the course of three major epidemics throughout history [8], with modern outbreaks still occurring, most recently in Madagascar [9]. One of the factors responsible for the virulence of this organism is its ability to avoid phagocytosis by macrophages [10]. This ability is dependent upon a gel-like capsule, composed of polymers of the capsular antigen fraction 1 (Caf1) protein, which coats the bacterium and is thought to act by preventing adhesin-receptor interactions. Deletion of the capsule causes an increase in the uptake of *Y. pestis* bacilli by macrophages [10] showing Caf1 to be an important protection against the host’s immune system.

Caf1 forms very long, thin polymers consisting of repeating ~ 15 kDa subunits with polymer lengths of up to 1.5 μm having been observed [11]. The polymers are highly flexible, appearing at high magnifications like beads on a string. Their biogenesis proceeds via the chaperone-usher (CU) pathway, which is employed for the production of a range of pilus structures in Gram negative bacteria [12]. Briefly, nascent, unfolded Caf1 subunits are exported to the periplasm, where they are bound by the chaperone protein Caf1M. This partially stabilises the Ig-like fold of the Caf1 subunit, before it is delivered to the usher protein, Caf1A, which resides in the outer membrane. Caf1A assembles the Caf1 subunits into a polymer that it exports to the cell surface [12] where it appears as a diffuse “flocculent” layer above the pellet in centrifuged cultures [13, 14]. The molecular basis for the polymerisation is the donation of the N-terminal β strand of one subunit into the acceptor cleft of the preceding subunit in the polymer, displacing the chaperone and stably completing the subunit fold [15]. This process, known as donor-strand complementation, results in a highly stable, non-covalent polymer [16].

In *Y. pestis*, Caf1 is expressed from the *caf1* operon present on the pMT plasmid [17]. Upstream of the *caf1M*, *caf1A* and *caf1* genes of the *caf1* operon is a fourth gene, *caf1R*, which is encoded on the opposite DNA strand. The domain structure of the Caf1R protein is that of a Rob-like AraC family transcription factor [18], consisting of a recognisable N-terminal helix-turn-helix DNA binding domain but with a distinctive C-terminal domain of unknown function. Although a putative promoter site and regulatory elements have been predicted upstream of *caf1M* [19], their presence has yet to be empirically determined.

*Y. pestis* becomes vulnerable to macrophage attack when it is injected into the mammalian host via a flea bite. The bacterium senses this event through a range of molecular mechanisms that perceive the temperature of its environment and exploit the temperature difference between the flea (on the hosts surface ~ 25 °C) and blood (~ 37 °C) [8]. Caf1 protein expression is induced at 37 °C [8, 10, 20], allowing the bacteria to rapidly produce this protein when most at risk of being phagocytosed. Temperature dependent increases in *caf1* operon gene transcription have been reported [20, 21] but whether Caf1R plays a role in coordinating the correct temperature dependent expression is not clear.

A previous report has described the Caf1R protein as a positive transcriptional regulator [22]. There are three arguments in favour of this designation: first, a plasmid containing only the 81 N-terminal residue helix-turn-helix DNA binding domain of Caf1R caused an increase in Caf1 production [22, 23]; second, inducing frame-shift mutations into Caf1R prevented Caf1 biogenesis; and finally, Caf1R displays homology with the AraC family of positive transcriptional regulators [22]. However, a link between Caf1R and the thermal switch has not been demonstrated.

Here, we determine the role of Caf1R in the thermosensitive control of gene expression from the *caf1* operon. We show that the *caf1M*, *caf1A* and *caf1* genes are transcribed as a single polycistronic transcript and identify the promoter region that is critical for their transcription. Deletion of *caf1R* completely abrogated Caf1 polymer expression, which was restored by complementing this deletion with *caf1R* on a second plasmid. Finally, RT-PCR and western blot experiments showed *caf1* operon transcripts are substantially upregulated upon a switch from 25 °C to 35 °C in a Caf1R dependent manner. Therefore, we propose that the temperature induced expression of Caf1 polymers is governed at the transcriptional level in a Caf1R dependent manner, thus revealing Caf1R to be a key factor in the determination of *Y. pestis* virulence.

## Results

### The *caf1* operon is transcribed as a polycistronic transcript

**Fig. 1:**
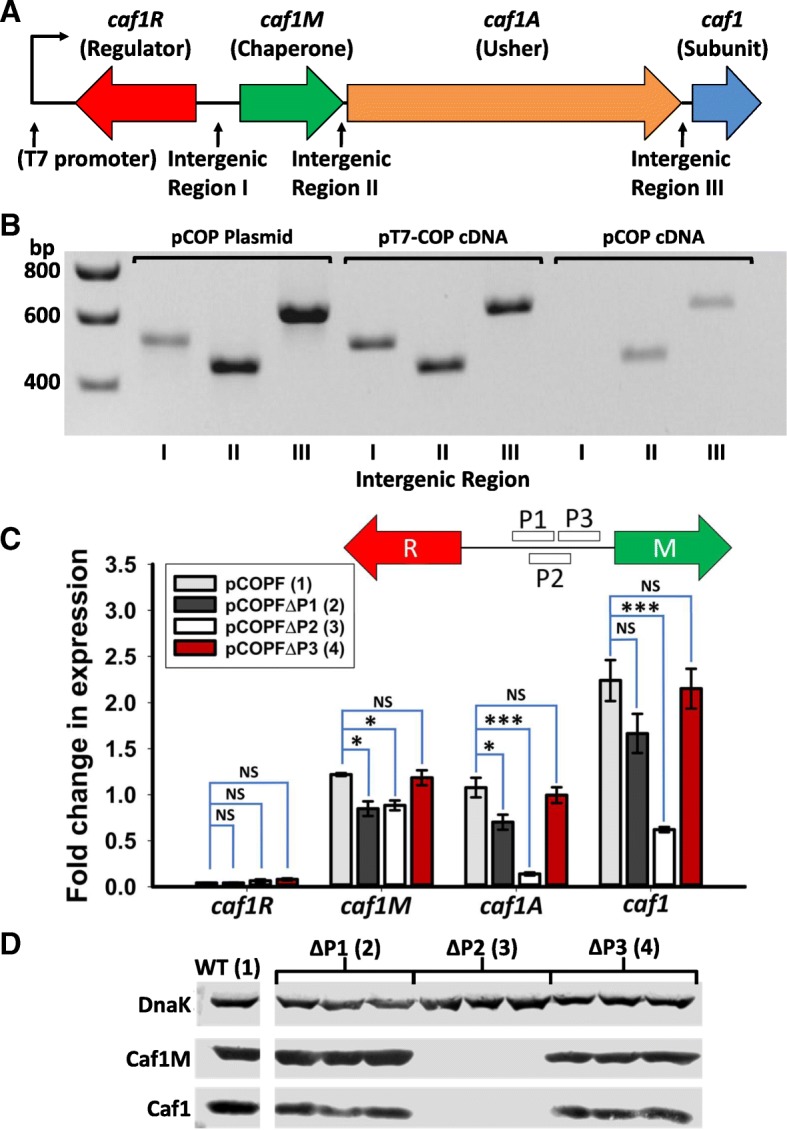
Determination of *caf1* transcript size and promoter site. **a** Organisation of the *caf1* operon. The position of the T7 promoter, found in the artificial pT7-COP plasmid is highlighted. **b** Agarose gel showing the PCR amplification products corresponding to the three intergenic regions of the *caf1* operon generated from either the pCOP plasmid, or cDNA made using mRNA from cultures of *E. coli* transformed with pT7-COP or pCOP plasmids and grown at 35 °C for 16 h. Both plasmids contain the *caf1* operon as shown in (**a**). **c** Transcript levels of the *caf1* operon genes as determined by RT-PCR, from cultures grown for 16 h of *E. coli* transformed with either pCOPF (full *caf1* operon, Caf1R,M and A have FLAG tags), or pCOPF with the proposed promoter regions P1,2 or 3 deleted. Three cultures of each condition were grown, with RT-PCR reactions run in duplicate for each culture. Bar heights correspond to mean fold-change in expression relative to β-lactamase. Error bars represent standard error of the mean (S.E.M) from three biological replicates. Asterisks represent significant differences between groups (* - *P* < 0.05, ** - *P* < 0.01, *** - *P* < 0.001, NS – not significant, determined by ANOVA with Holm- Šidák *post-hoc* test). A diagram detailing the positions of the P1, 2 and 3 regions is shown in the top right of the graph. **d** Western blot of the above cultures showing the levels of Caf1M and Caf1 (detected by anti-FLAG tag and anti-Caf1 antibodies respectively), and using DnaK (detected by an anti-DnaK antibody) as a loading control

In the *caf1* operon, the *caf1R* coding sequence is on the opposite strand to, and separated by a 328 nucleotide (nt) intergenic region I, from the remaining genes, which are arranged with a very short (24 nt) intergenic region II between *caf1M* and *caf1A* and a larger intergenic region III of 80 nt between *caf1A* and *caf1* (Fig. 1a). To characterise the expression of *caf1* genes within the *caf1* operon, PCR analysis of the RNA transcripts was employed. Cultures of *E. coli* transformed with a plasmid containing the *caf1* operon (**pC**af1**OP**eron, pCOP, Additional file 1: Table S1) or the operon preceded by an upstream T7 promoter from the vector (pT7-COP, Additional file 1: Table S1), were grown from single colonies for 16 h at 35 °C to stimulate Caf1 production, following which total RNA was extracted and used to synthesise cDNA. To assess whether the *caf1* genes are expressed as part of a polycistronic transcript, the cDNA was used as a template, with primers complementary to the coding regions either side of the intergenic regions (Additional file 1: Figure S1). Therefore, if the genes are expressed on a large transcript, amplification of the intergenic regions should proceed when the cDNA is used as a template, whereas single transcripts will result in no amplification. Amplification products could be detected from intergenic regions II and III from pCOP, showing that *caf1M* and *caf1A*, as well as *caf1A* and *caf1*, are co-transcribed (Fig. 1b). The intergenic region between *caf1M* and *caf1A* is very small (24 nt), and unlikely to harbour a promoter. This suggests that in the natural system, *caf1M*, *caf1A* and *caf1* are all transcribed together on one mRNA of approx. 3900 nt. For pT7-COP, all three intergenic regions could be detected, suggesting that the T7 promoter, which is upstream of *caf1R*, drives transcription that proceeds through all four genes. This was of interest since we had previously found by chance that leaky expression from this T7 promoter was able to drive significant Caf1 polymer production [13].

### Identification of the long *caf1* operon transcript promoter

To identify the promoter that drives transcription of this long mRNA, the DNA sequence between *caf1R* and *caf1M* (intergenic region I) was used as an input into the BPROM (Softberry Inc., Mount Kisco, NY, USA, http://www.softberry.com) [24] and Neural Network Promoter Prediction 2.2 (NNPP2.2) [25] webservers. These predictions led us to define three potential promoter regions of 41 bp (P1, P2 and P3, Additional file 1: Figure S2) upstream of *caf1M* that could potentially drive the transcription of the polycistronic *caf1* transcript. Interestingly, the P2 region contains the putative promoter previously predicted by Galyov et al. [23]. Each region was then separately deleted from pCOPF (in which Caf1R, M and A have added C-terminal FLAG tags, Additional file 1: Table S1) and the resulting plasmids used to transform BL21(DE3) cells. To assess the effect of the deletions, protein levels after 16 h of growth at 37 °C were determined by western blot using anti-FLAG antibodies and levels of each transcript were measured by RT-PCR (Fig. 1c,d). Deletion of the P3 region showed little effect on the levels of protein or transcript. For P1, deletion had little effect on protein expression, but did appear to cause a small drop in transcript level. However, the greatest effect was seen upon deletion of the P2 region, where synthesis of all the Caf1 proteins was completely abrogated, accompanied by a substantial decrease in *caf1A* and *caf1* transcript levels. The levels of c*af1M* transcript in the P1 and P2 deletions are both slightly reduced, but whereas Caf1M synthesis is unaffected by P1 deletion, no Caf1M protein was detected from the P2 delection. The deleted P2 region thus contains or overlaps the promoter responsible for the large, polycistronic transcript observed by PCR analysis.

### Caf1R regulates the *caf1* operon

**Fig. 2:**
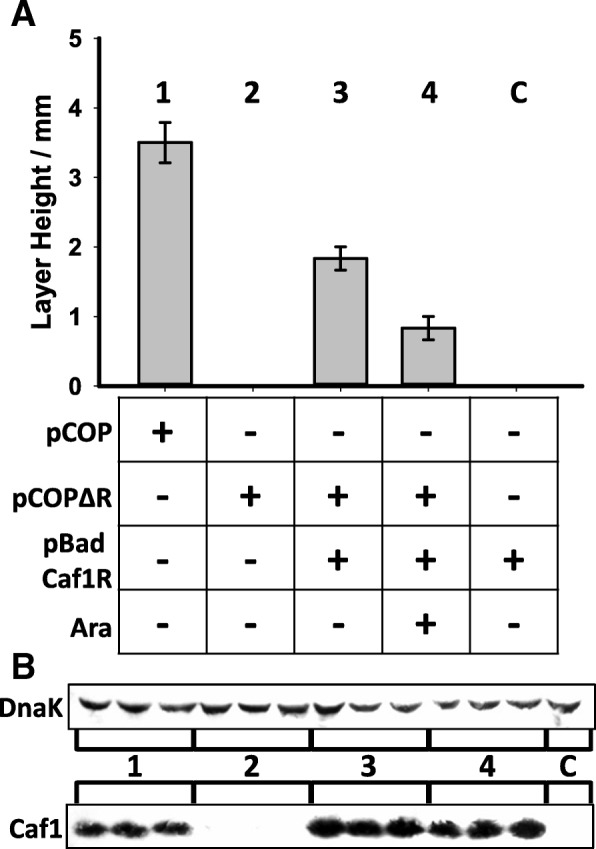
Caf1 protein production is dependent upon low levels of Caf1R. **a** Graph of the flocculent layer height obtained from *E. coli* cultures containing either pCOP (full *caf1* operon, 1), pCOPΔR (*caf1* operon lacking Caf1R, 2), a co-transformation of pCOPΔR and pBad Caf1R (arabinose inducible Caf1R) without (3) and with (4) 0.005% *w*/*v* arabinose added to the culture, and pBad Caf1R only control (C). Cultures were grown for 16 h at 35 °C. Bar heights correspond to the mean flocculent layer height obtained from three separate cultures where error bars represent the standard error of the mean (S.E.M). **b** Western blot of three separate cell pellets from the above cultures, probed with an anti-Caf1 antibody. DnaK was used as a loading control, probed with an anti-DnaK antibody

To further investigate the importance of Caf1R to Caf1 polymer production, the c*af1R* gene was deleted from the pCOPF plasmid (pCOP∆R, Additional file 1: Table S1). The pCOPF and pCOP∆R plasmids were transformed into BL21(DE3) *E. coli* cells and expression cultures were grown from single colonies at 35 °C. Expression of Caf1 polymers correlates with the appearance of a flocculent layer that sits on top of the cell pellet in centrifuged expression cultures [13, 14]. Expression cultures were assayed for flocculent layer production, which directly corresponded to the amount of Caf1 polymer secreted by the bacteria (Additional file 1: Figure S3). The *caf1R* deletion completely abolished Caf1 protein production from the pCOP∆R plasmid, with no flocculent layer observed and no Caf1 protein detectable in the pellet by western blot (Fig. 2), demonstrating that *caf1R* is necessary for Caf1 production.

pCOPF∆R was then co-transformed into BL21(DE3) cells with pBad-Caf1R (Additional file 1: Table S1), where *caf1R* is under the control of an arabinose inducible promoter. Complementing pCOPF∆R with pBad-Caf1R in the absence of arabinose rescued Caf1 protein expression (Fig. 2). Induction of Caf1R from the pBad Caf1R by the addition of arabinose also rescued Caf1 protein expression, although the height of the flocculent layer was lower in this case suggesting that only very low levels of Caf1R are needed to promote Caf1 protein expression, and that higher levels are inhibitory to Caf1 biogenesis. These results show that Caf1R is essential for Caf1 production.

### Temperature induction of *caf1* operon expression occurs at the transcriptional level

It is useful for *Y. pestis* to control the formation of the Caf1 polymer using temperature in order to provide immediate protection from macrophages upon transfer to a warm blooded host. The increase in *caf1* operon expression with temperature has been demonstrated previously [8, 20, 21], but the underlying molecular mechanism has not been investigated. Therefore, we tested the hypothesis that the Caf1R protein is the transcription factor that mediates the thermoregulation of Caf1 expression.

**Fig. 3:**
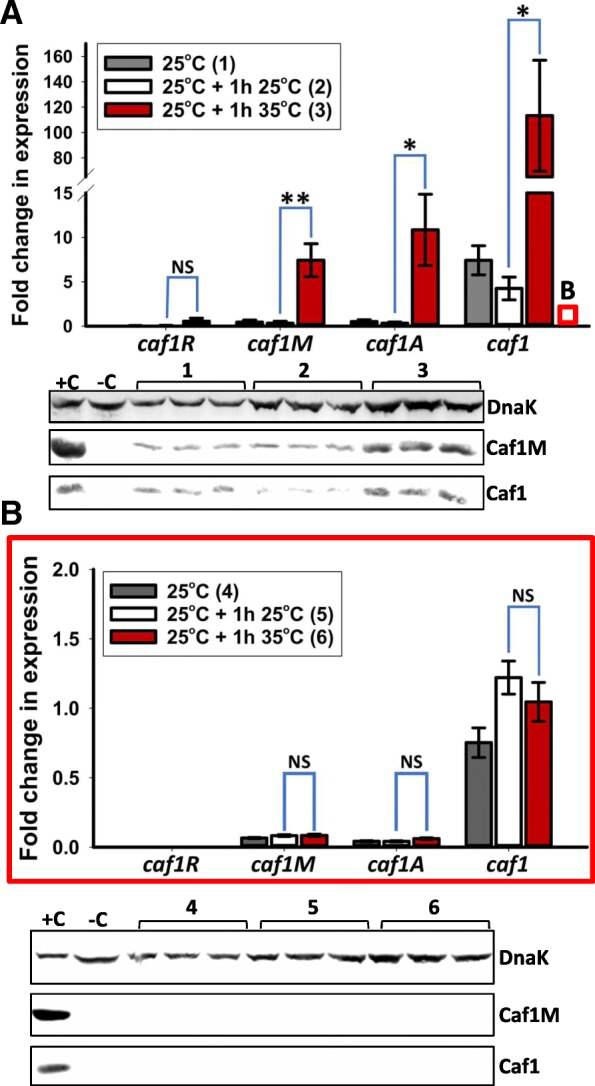
*caf1* operon gene expression is temperature and Caf1R dependent. Transcript levels, determined by RT-PCR, of each *caf1* gene are shown for cultures of *E. coli* transformed with pCOPF (full *caf1* operon, (**a)**) and pCOPFΔR (*caf1* operon, Caf1R deleted, (**b)**), grown at 25 °C overnight (~ 16 h), then either 25 °C or 35 °C for 1 further hour. The red box in **a** shows the approximate scale of the Y-axis in **b**. Three cultures of each condition were grown, with RT-PCR reactions run in duplicate for each culture. Bar heights correspond to mean fold-change in expression relative to β-lactamase. Error bars represent standard error of the mean (S.E.M) from three biological replicates. Asterisks represent significant differences between groups (* - P < 0.05, ** - P < 0.01, *** - P < 0.001, NS – not significant, determined by ANOVA with Holm- Šidák *post-hoc* test). Western blots showing the levels of Caf1M and Caf1 (detected by anti-FLAG tag and anti-Caf1 antibodies respectively) in the cell pellets of the expression cultures are displayed underneath each graph, using DnaK probed with an anti-DnaK antibody as a loading control. +C and –C represent the pellets of BL21(DE3) cells transformed with pCOPF and untransformed respectively, and grown for 16 h at 35 °C

To assess this claim, *E. coli* were transformed with pCOPF or pCOPF∆R and grown for 16 h at 25 °C, such that *caf1* is not expressed. The stationary phase cultures were then diluted to 0.5 OD_600_ and then grown for one further hour at either 25 °C or 35 °C. Transcriptional levels of the *caf1* operon genes were then examined through RT-PCR. A large, significant increase in the relative transcript levels of *caf1M*, *caf1A* and *caf1* could be seen at 35 °C compared to 25 °C for cells transformed with pCOP, with fold increases of 23.3, 33.4 and 26.7 respectively (Fig. 3a). An 8.8 fold increase in *caf1R* transcript levels was also observed, but this was not statistically significantly different from the levels observed at 25 °C. Crucially, in the absence of Caf1R, no temperature dependent increase was seen (Fig. 3b), showing that the increase in transcription levels was not a general effect of temperature change. Additionally, the overall level of transcript produced from pCOPF∆R was significantly lower in all cases when compared to the system containing all four genes, again highlighting the influence of Caf1R.

The effect of the temperature change was then analysed at the translational level by performing western blot experiments on the same cultures used for RT-PCR. The levels of protein detected matched the RT-PCR data, where levels of Caf1M and Caf1 protein could be seen to increase upon incubation at the higher temperature relative to the lower temperature, while being completely undetectable in the absence of Caf1R (Fig. 3). Caf1A, a large membrane protein, was not visualised by western blot. Similarly, although Caf1R is soluble and of a similar size to the easily detected Caf1M, it was also not detected by anti-FLAG western blot, presumably in this case because its concentration was consistently below the limit of detection. This is not surprising, as transcription factors are often present in very low amounts in bacterial cells [26, 27], and our data show that even the low levels of expression from the uninduced, tightly regulated P_BAD_ promoter [28] are enough to rescue Caf1R deletion (Fig. 2).

**Fig. 4:**
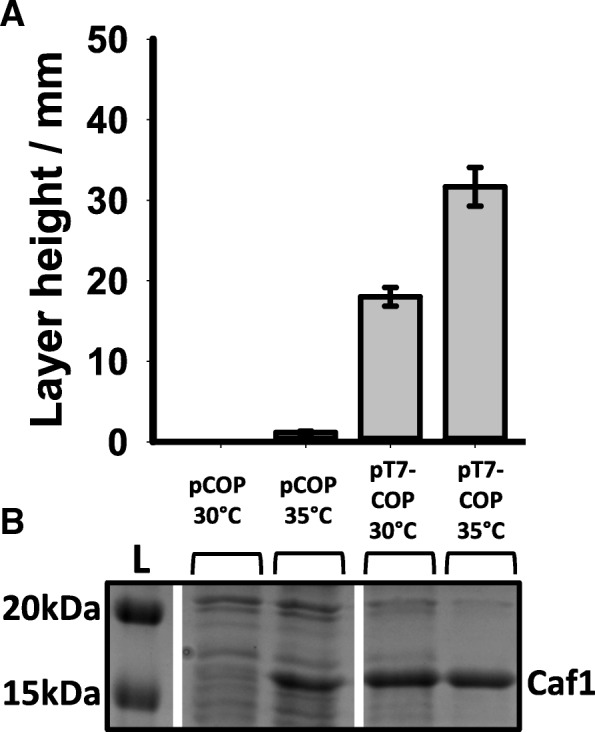
Effect of a T7 promoter on Caf1 production at 30 °C and 35 °C. *E. coli* cells were transformed with either pCOP (full *caf1* operon) or pT7-COP (T7- full *caf1* operon) and grown at either 30 °C or 35 °C for 16 h, after which the flocculent layer height was measured (**a**) and analysed by SDS-PAGE (**b**). Error bars represent standard error of the mean (S.E.M) from three replicate cultures. The position of the band corresponding to Caf1 is highlighted next to the gel

When *E. coli* transformed with the pT7-COP plasmid were grown at 30 °C (where the natural Caf1R system is switched off and transcription is driven by the T7 promoter), high levels of Caf1 expression could still be detected (Fig. 4). The T7 promoter appears to be stronger than the natural *caf1R* promoter, since it generates a greater amount of flocculent layer. Differences in flocculent height for pT7-COP containing cells are likely due to differences in the efficiency of T7 transcription between the two temperatures [29], and also the thermal activation of *caf1* operon transcription from the native promoter at 35 °C. The relative thermal independence of the T7 driven expression implies that the temperature regulation of the *caf1* operon takes place primarily at the transcriptional level, with post-transcriptional (translational and post-translational) regulation having very little, if any, role.

## Discussion

### *caf1* operon genes are expressed from a polycistronic transcript

The results of this study show that the *caf1* operon is expressed as a long, polycistronic transcript from a promoter that resides in the P2 region in a Caf1R dependent manner. Previously, Galyov et al. predicted the presence of regulatory regions and a putative promoter that would drive *caf1M *transcription, and identified a Shine-Dalgarno sequence 7 bases upstream of the Caf1M start codon [23]. We used an independent software tool that identified potential promoter regions that could transcribe the polycistronic *caf1* operon transcript, before confirming their activity through deletion mutagenesis and RT-PCR. The putative promoter predicted by Galyov et al. overlaps with the P2 region we identify here as being essential for the expression of the operon. Therefore, we have independently identified and experimentally confirmed the presence of the promoter responsible for transcription of the *caf1* operon.

Considering *caf1M*, *caf1A* and *caf1* are transcribed as a single polycistronic transcript, it was surprising that the RT-PCR results showed that each of the genes has a particular transcriptional level, where the levels of *caf1M* and *caf1A* transcripts were similar and *caf1* transcript much higher. There are two potential explanations of this: either the *caf1* gene can be transcribed by a second, Caf1R responsive promoter, or the translational units within the polycistronic RNA transcript have different levels of mRNA stability. We used 5′ RACE (**R**apid **A**mplification of **c**DNA **E**nds) to search for a second promoter upstream of the *caf1* gene. If a promoter exists in this region, the majority of transcript sequences should terminate inside intergenic region III at the transcriptional start site (Additional file 1: Figure S4), but 7 out of 8 sequences obtained continued through the intergenic region into *caf1A* (Additional file 1: Figure S5), thus corresponding to the observed polycistronic transcript. These sequences terminated at different locations, implying the termination is due to dissociation of the polymerase enzyme rather than a transcriptional start site within this region of *caf1A*. Moreover, deletion of the P2 region, which lies upstream of *caf1M* and is therefore responsible for transcription of the polycistronic transcript, abolished the expression of all *caf1* genes. If separate promoters were present for each gene, this deletion would have a substantially reduced effect on *caf1A* and *caf1* expression. A previous study by Galyov et al. [19] predicted the presence of a putative promoter upstream of *caf1*, at the 3′ end of the *caf1A* gene. However, in isolation this region did not enable *caf1* expression [19], suggesting that it does not contain a functional promoter, in concordance with our results.

As a second promoter could not be detected, the differential stability of the translational units within the mRNA, such as has been previously reported [30], is a more plausible explanation for the differing levels of transcript observed in the RT-PCR experiments. Upon a shift of the bacteria to 35 °C, such a system would allow Caf1R to activate the transcription of the *caf1* operon polycistronic mRNA, which would then be degraded to different degrees resulting in differential levels of transcript for *caf1M*, *caf1A* and *caf1*. To produce long Caf1 polymers, a large number of subunits is required, with fewer chaperones and ushers necessary. Therefore, this system appears to allow cells to produce the optimal levels of each protein from a single transcript.

### Thermosensitive transcriptional regulation of the *caf1* operon

The ability to regulate gene expression in response to changes in temperature is important for *Y. pestis*, which lives mainly in two environments with different ambient temperatures: the flea at ~ 25 °C [8], and mammals, with body temperatures of around 37 °C. This difference in temperature is therefore a simple signal that allows the bacterium to determine whether it is in the vector or the host where it behaves as a mammal pathogen, expressing factors related to virulence and immunoevasion. The results of the temperature switching experiment clearly show the rapid upregulation of the expression of *caf1M, caf1A* and *caf1* upon an increase in temperature. The fold increase in transcript levels with temperature, that we observe, correlates well with those previously described [20] for *caf1R* and *caf1M* (8.8 vs 8.9 fold and 23.3 vs 19.7 fold respectively), but we detect greater increases in transcription for *caf1A* and *caf1* (33.4 vs 9.7 fold and 26.7 vs 7.8 fold respectively). Crucially, this upregulation occurs at the transcriptional level, and is wholly Caf1R dependent.

The difference in transcriptional output is sufficient to describe the observed temperature sensitivity of Caf1 protein production. Not only did the levels of Caf1M and Caf1 observed by western blot correlate with the levels of transcript seen by RT-PCR but bypass of transcriptional regulation through the introduction of a T7 promoter permitted expression of Caf1 at 30 °C, when there was no expression from the native *caf1* operon under these conditions. If a method of translational or post-translational regulation was present (e.g. a riboswitch), then it would be expected that these mechanisms would regulate T7 driven *caf1* expression at lower temperatures. Therefore, these mechanisms are either not present, or have a very weak effect in the Caf1 system. Caf1R mediated transcriptional control is thus the main mechanism to express Caf1 polymers under the appropriate conditions.

### Caf1R mediates thermal regulation

**Fig. 5:**
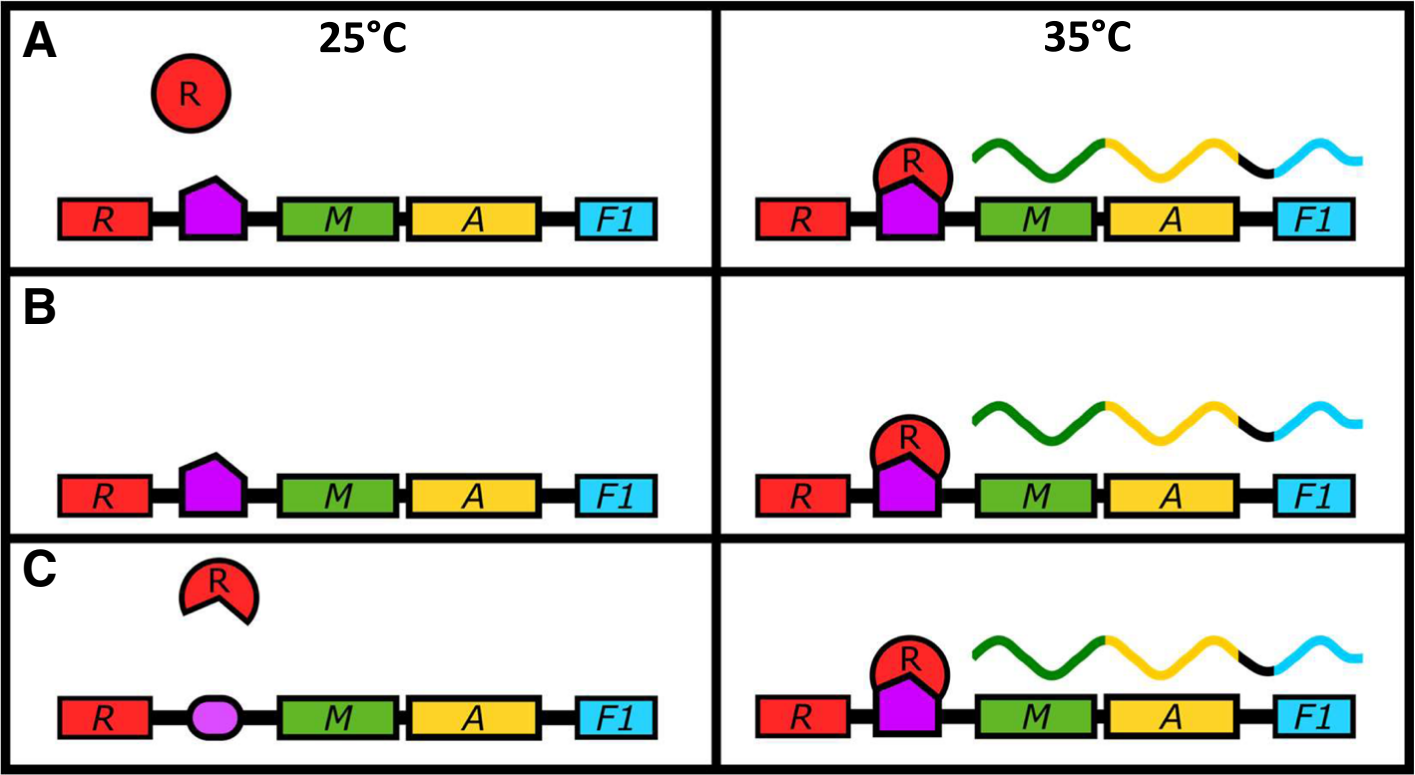
Models of thermoresponsive Caf1R-dependent transcriptional control of gene expression. Three models that potentially describe the Caf1R dependent thermoresponsive increase in *caf1* operon transcription are shown, with their states at 25 °C and 35 °C depicted in the left and right hand side panels respectively. In model (**a**), the Caf1R protein is the thermoresponsive element. At 25 °C it is unable to activate transcription of the *caf1* operon, regardless of its DNA binding state. At 35 °C, a conformational change allows the protein to activate transcription. Inactive Caf1R is shown as a red circle and active Caf1R as a red arc. In (**b**), the Caf1R protein abundance is thermoresponsive. Transcription is not thermally regulated so either Caf1R is always transcribed but only translated at 35 °C or Caf1R is stabilised at 35 °C. The increased Caf1R protein level activates transcription of the operon. In (**c**), the DNA is the thermoresponsive element. At 25 °C, the DNA is not in an optimal conformation for Caf1R binding, and so transcription is not activated. At 35 °C, a change in the DNA facilitates optimal Caf1R binding, and so transcription of the operon is activated. Genes are shown as coloured rectangles (red, green, yellow and blue for *caf1R*, *caf1M*, *caf1A* and *caf1* respectively), with the Caf1R DNA binding site shown in purple. mRNA transcripts are represented as wavy lines coloured according to the gene they are transcribed from

It is clear from our results that Caf1R is the element that transduces the temperature change into a transcriptional response. There are three models through which this could take place (Fig. 5). In model A (Fig. 5a), the Caf1R protein itself is the thermoresponsive element. At 25 °C the protein is either unable to bind DNA, or unable to stimulate transcription of the *caf1* operon. Upon a shift in temperature to 35 °C, the protein changes conformation, becoming active and inducing transcription of the *caf1* operon. In model B (Fig. 5b), the thermoresponsive element is the level of Caf1R, mediated either through temperature dependent increase in *caf1R* translation or decrease in Caf1R protein turnover. Finally, in model C (Fig. 5c), a temperature dependent structural change in a DNA regulatory region, e.g. supercoiling or promoter topology [1] is recognised by Caf1R, which binds and activates transcription.

Well studied examples found in *Yersinia* show how transcription factors can be thermally regulated in very different ways. In one case LcrF, which controls Type III secretion systems (TTSS) involved in phagocytosis evasion, undergoes translational control through the use of an RNA thermometer in which ribosome binding to the LcrF mRNA is inhibited by a temperature sensitive RNA secondary structure [2]. In contrast, the RovA transcription factor is itself a proteinaceous thermosensor. RovA functions as a “global regulator” of transcription in response to increases in temperature, resulting in the expression of the TTSS and Yop effector proteins in *Y. pestis*, and the internalization factor invasin in *Y. pseudotuberculosis* [31]. The mechanism through which RovA senses temperature involves a flexible loop situated between two alpha helices that have a role in dimer formation [7]. Upon temperature increase, the protein partially unfolds, lowering its affinity towards DNA and increasing its susceptibility to proteases, thus de-repressing the transcription of target genes.

The closest homologue of Caf1R is the Rob protein of *E. coli* [18], which consists of an N-terminal DNA binding domain coupled to a C-terminal GyrI-like domain [32]. Rob acts as a transcriptional activator that is induced by dipyridyl or decanoate [33]. In the uninduced state, the C-terminal domain causes sequestration of Rob into distinct cellular foci, preventing it from interacting with DNA and activating transcription. Addition of the inducer allows Rob to disperse through the cell and activate the transcription of its target genes [33] though direct interactions between its N-terminal domain and the σ^70^ subunit of RNA polymerase [34]. Truncated Rob proteins, where the GyrI domains were absent, were able to activate transcription in the absence of inducer [33, 35]. Additionally, MarA, which is homologous to the N-terminal DNA binding domain of Caf1R, contains no C-terminal domain, and so regulates transcription on the basis of its abundance rather than the presence of an inducer [33, 36]. A truncated Caf1R protein, containing only the first 82 amino acids of the DNA binding domain, was seen to allow expression of Caf1, whereas complete Caf1R knockout prevents Caf1 expression [22, 23]. The homology of Caf1R to these proteins therefore suggests that its N-terminal DNA binding domain is responsible for binding DNA and activating transcription, whilst the C-terminal GyrI-like domain is responsible for the characteristic Caf1R behaviour. Whether this is due to changes in Caf1R conformation (model A) or abundance (model B) we cannot resolve. Additionally, model C would also account for the results we describe here and Karlyshev et al. observed a series of repeated DNA sequences within intergenic region I which may interact with Caf1R and orient the DNA for optimal induction of gene expression [22]. It is also possible that a combination of these models is responsible. We made numerous attempts to purify sufficient amounts of Caf1R for such studies, but it appeared to be particularly unstable. Therefore, efforts to produce a soluble, functional construct will likely be necessary in order to answer these questions.

### Thermosensitive transcriptional activators in Gram-negative bacterial pathogens

The function of Caf1R revealed in this study is of interest due to its role in the immunoevasive ability of a highly dangerous pathogen. However, it is possible that Caf1R also represents a class of transcription factors that has received little prior study. PSI-BLAST searches reveal that the most similar proteins to Caf1R are unannotated sequences from various Gram-negative bacteria such as *E. coli* and several *Salmonella* species. However, one similar protein sequence is LdaA from an enteropathogenic *E. coli* strain (EPEC). LdaA is a putative regulatory protein that exists as part of the *lda* locus, named locus of diffuse adherence for its apparent ability to attenuate the adherence of *E. coli* to HEp-2 cells [37]. The *lda* gene displays sequence similarity with other fimbrial proteins, particularly the K88 Fae proteins, but unlike these structured fimbriae, Lda fibres appear flexible when visualized by electron microscopy. These data, as well as the similarity of LdaE to known periplasmic chaperones [37], hint that the Lda proteins may assemble in a manner similar to Caf1, and form a similar structure. If this is the case, the sequence similarity of LdaA and Caf1R suggests it may potentially function in an analogous way. Additionally, homology between Caf1R and AfrR, a putative transcription factor in the AF/R1 operon, has been identified previously [38]. The AF/R1 pilus produced by the genes of this operon plays an important role in the adhesion and pathogenicity of a rabbit diarrheagenic *E. coli* (RDEC) strain. If these proteins indeed function in a similar way to Caf1R, it may be evidence of a conserved system of thermosensitive transcriptional activation present in Gram-negative pathogens.

## Conclusion

Thermosensitive expression of the *caf1* operon is determined on the transcriptional level in a Caf1R dependent manner. The *caf1* operon genes *caf1M*, *caf1A* and *caf1* are transcribed as a single polycistronic mRNA, from a promoter within the P2 region upstream of *caf1M*. The transcription of this mRNA is substantially upregulated upon a temperature shift from 25 °C to 35 °C. This is sufficient to allow protein expression and the subsequent formation of Caf1 polymers. As Caf1 has a key role in allowing *Y. pestis* bacteria to evade phagocytosis, these results reveal a simple but important mechanism through which a dangerous pathogen can sense and respond to its environment.

## Methods

### Plasmids and cell culture

Plasmid constructs (Additional file 1: Table S1) were derived from pGEM-T Caf1 and pBad33SD Caf1, described previously [13]. pCOP was constructed by substitution of the T7 promoter with a random sequence of the same length using a Q5 Site-directed mutagenesis kit (New England Biolabs). All other plasmids were constructed using an In-Fusion HD cloning kit (Clontech). Deletions were made by omitting the relevant region of the plasmid from the amplified region, then ligating the amplified region back together. Primers sequences are shown in Additional file 1: Table S2.

Transformations were performed using *E. coli* BL21 (DE3) (New England Biolabs). Three different colonies were selected from each plate and grown in LB media overnight at 37 °C, 180 rpm. Glycerol stocks were prepared for all transformed bacteria using 500 μl of bacterial culture and 500 μl of 60% (*v*/v) glycerol solution. Expression cultures were prepared using 5 ml TB media containing 100 μg ml^− 1^ ampicillin and/or 20 μg ml^− 1^ chloramphenicol and were inoculated using a stab from one of the glycerol stocks. To induce expression from pBad plasmids, L-arabinose was added at the concentrations described in the text at the same time as the cultures were inoculated. The flocculent layer heights were measured using a ruler after the centrifugation of capillary tubes containing Caf1 expression cultures at 2367 x g, 22 °C for 15 min.

For analysis of *caf1* operon transcript levels, bacterial cells were cultured in triplicate from glycerol stocks of *E. coli* BL21 (DE3) cells transformed with pCOPF (Additional file 1: Table S1) and grown at 25 °C overnight. The OD_600_ was measured for all cultures and then 2 ml were taken from each culture to be analysed by RT-PCR and western blot. The remaining cultures were diluted to an OD_600_ of 0.5 and then each culture was split into two cultures: one to be incubated at 25 °C and the other simultaneously at 35 °C. After one hour incubation the OD_600_ was measured and 2 ml samples taken from each culture for RT-PCR and western blot analysis.

### RNA extraction and cDNA synthesis

RNA was isolated using an EZ-10 Total RNA Mini-Preps Kit (Bio Basic Inc.) by transferring 1 ml from each culture (~ 2 × 10^9^ cells) separately to be centrifuged at 10,000 x g for 1 min. Supernatants were discarded and 100 μl of lysozyme solution (400 μg ml^− 1^ lysozyme in RNase-free water) added to each sample pellet. The mixtures were resuspended thoroughly and RNA extracted according to the manufacturer’s protocol. The concentration of RNA was measured using a Nanodrop UV spectrophotometer (Labtech). RNA samples were then used to perform cDNA synthesis using a QuantiTect Reverse Transcription Kit (Qiagen) according to the manufacturer’s protocol. The resulting concentration of cDNA was measured using a Nanodrop UV spectrophotometer and the samples stored at -20 °C.

### PCR and RT-PCR

The master reaction mix for RT-PCR experiments was prepared using GoTaq Flexi DNA Polymerase (Promega) by adding 4 μl nuclease-free water, 4 μl 5X Colorless GoTaq Flexi Buffer, 3 μl of 2 mM dNTPs, 3.2 μl of 25 mM MgCl_2_, 0.2 μl SYBR Green (Sigma, S9430) (diluted 200 times with 100% DMSO), 0.2 μl GoTaq Flexi DNA Polymerase, 0.8 μl 10 μM primer mix and 5 μl of 50 ng μl^− 1^ cDNA per reaction. Samples were loaded in a Rotor-Gene Q instrument (Qiagen) and critical threshold cycle values (CT values) were collected to measure the fold change in gene expression for each target gene, relative to β-lactamase transcription from the plasmid, with a threshold level of 0.5. Primer sequences are shown in Additional file 1: Table S2, with a schematic of the design shown in Additional file 1: Figure S1. Thermal cycling conditions are stated in Additional file 1: Table S3.

For transcript analysis, PCR was conducted using Phusion High-Fidelity DNA Polymerase kit from (New England Biolabs) by adding 10 μl of 5X Phusion GC buffer, 1 μl of 10 mM dNTPs, 2.5 μl each of 10 μM forward and reverse primers, 2 μl of 10 ng μl^− 1^ template DNA, 1.5 μl of DMSO, 0.5 μl of Phusion DNA polymerase and nuclease-free water up to 50 μl. Thermal cycling conditions are stated in Additional file 1: Table S4.

### Rapid amplification of cDNA ends (RACE)

5′ RACE experiments were performed as described previously [39] with minor modifications. Briefly, cDNA was prepared by performing the reverse transcriptase reaction using a 9 bp long gene specific primer (Additional file 1: Table S2) complementary to a 3′ region of the *caf1* gene. cDNA samples were diluted two times with nuclease-free water and purified using 10 kDa molecular weight cut-off Vivaspin centrifugal concentrators (Sartorius) spun at 1000 x g for 30 min. This step was repeated to prepare the samples for poly-adenine tailing.

Poly-adenine tailing was performed by mixing 0.5 μl terminal transferase enzyme (20 Units / μl, New England Biolabs), 5 μl 10 x terminal transferase buffer, 5 μl of 2.5 mM CoCl_2_, 0.5 μl of 10 mM dATP for tailing,, 5 pmol of cDNA to be tailed and nuclease-free water up to 50 μl. The mixture was incubated at 37^o^ C for 30 min and then heated at 70^o^ C for 10 min to inactivate the reaction.

Tailed cDNA was then amplified by PCR. The PCR reaction was prepared by mixing 0.5 μl of 2 U/μl Phusion DNA polymerase (New England Biolabs), 10 μl 5 x GC Phusion buffer, 1 μl of 10 mM dNTP mix, 1.2 μl of 10 μM (dT)17-adapter primer (Additional file 1: Table S2), 2.5 μl of 10 μM adapter primer (Additional file 1: Table S2), 2.5 μl of 10 μM gene specific primer (Additional file 1: Table S2) and 3% DMSO with 2 μl of tailed cDNA and nuclease-free water up to 50 μl. Cycling conditions are stated in Additional file 1: Table S5.

The PCR product was analysed by agarose gel electrophoresis. Amplification products were extracted from the gel using a Monarch gel extraction kit (New England Biolabs) according to the manufacturer’s protocol. The concentration of pure DNA was measured using a Nanodrop UV spectrophotometer (Labtech) and sequenced by Eurofins Genomics.

### Western blot

Bacterial cultures were centrifuged at 20,000 x g for 5 min. Supernatants and flocculent layers were discarded and cell pellets resuspended in 100 μl per OD_600_ of 100 mM DTT, 2% *w*/*v* SDS. The samples were heated at 95 °C for 10 min and centrifuged at 20,000 x g for 10 min. 50 μl of this supernatant was mixed with 50 μl SDS loading buffer (2% w/v SDS, 0.1% w/v Bromophenol blue, 5 mM EDTA, 125 mM Tris pH 6.8, 15% *v*/v Glycerol and 1% v/v β-mercaptoethanol), boiled for 5 min and centrifuged at 20,000 x g for 5 min. The samples (10 μl) were resolved on 12% SDS-PAGE. Nitrocellulose membranes and blotting papers were soaked in CAPS buffer (10 mM CAPS pH 11) containing 20% methanol for 10 min. SDS-PAGE gels were soaked in the same buffer for 2 min. The blotting paper, nitrocellulose membrane and gel were assembled in a semi-dry blotter (Trans-Blot® SD semi-dry transfer cell, Biorad) and 18 V applied for 30 min. Blots were stained with Ponceau S solution (Sigma) for 10 min to view the efficiency of protein transfer and then rinsed with PBS buffer. The blots were blocked with TBS buffer (2.7 mM KCl, 38 mM Tris-HCl and 140 mM NaCl pH 8) containing 5% milk (from powder) at room temperature for 2 h and then rinsed with (1x) TBS buffer. The membranes were incubated for 4 h at room temperature or overnight at 4 °C with 2 ml 5% milk solution 1 μg ml^− 1^ anti-FLAG antibody (Sigma) in order to detect the FLAG-tagged proteins and 6.9 μg ml^− 1^ anti-Caf1 antibody (Abcam) for Caf1 protein detection. The blots were washed with (1x) TBS buffer two times for 5 min at room temperature then incubated for 4 h at room temperature or overnight at 4 °C with 2 ml TBS buffer containing 2.5–5 μg mL^− 1^ anti-mouse antibody conjugated with horseradish peroxidase (Sigma). The blots were washed twice for 15 min with TBS buffer at room temperature, covered with developing solution and incubated at room temperature for a few minutes with shaking. Developing solution was prepared by dissolving 50 mg 4-chloronaphthol in 10 mL methanol and mixed with 50 ml developing buffer (20 mM Tris-HCl, 140 mM NaCl and 1 mM Na_2_HPO_4_ pH 7.2) containing 60 μl hydrogen peroxide. The blots were dried in air and the images were taken using a gel documentation system (Gel DocTM XR+, Biorad).

### Bioinformatics

Promoter sequences were predicted using the BPROM (Softberry Inc., Mount Kisco, NY, USA, http://www.softberry.com) [24] and Neural Network Promoter Prediction 2.2 (NNPP2.2, 25] webservers. Sequence alignments were generated using the Clustal Omega [40] webserver and visualised using the ESPript [41] webserver (http://espript.ibcp.fr).

### Statistical analysis

Statistical analyses were performed by one-way analysis of variance (ANOVA). Statistically significant differences between groups were then identified using a Holm-Šidák *post-hoc* test.

## Additional file

Additional file 1: **Table S1** List of constructs used in this study. **Table S2** List of primers used in this study. **Table S3** Thermal cycling conditions for RT-PCR. **Table S4** Thermal cycling conditions for PCR. **Table S5** Thermal cycling conditions for 5′ RACE. **Figure S1** Schematic of primer design. A diagram of the *caf1* operon present in the plasmids used in this study is shown, with arrows corresponding to the forward and reverse primers and placed in the approximate position where they bind. Forward primers are shown on top of the genes and reverse primers shown beneath. (A) Primers used for RT-PCR are shown in red for *caf1R*, green for *caf1M*, orange for *caf1A* and blue for *caf1*. (B) Primers used for detecting the presence of intergenic regions in cDNA are shown: purple for intergenic region I, olive for intergenic region II and dark blue for intergenic region III. **Figure S2** Schematic of the P1, P2 and P3 regions. The sequence of intergenic region I (INT1), located between *caf1R* and *caf1M* is shown, highlighted in green, with the P1, P2 and P3 regions aligned and highlighted in red, yellow and orange respectively. The ATG nucleotides corresponding to the start codon of *caf1M* are labelled with blue triangles. **Figure S3** Analysis of Caf1 content in the flocculent layer (A) Image of *E. coli* cultures containing the pT7-COP plasmid grown at 35 °C for the amounts of time stated, and centrifuged in capillary tubes to visualise the flocculent layer height. (B) SDS-PAGE analysis of the flocculent layers of the cultures from (A). (C) Graph of Caf1 band intensities in arbitrary units, obtained by densitometry of the gel shown in (B). **Figure S4** Diagram depicting 5’RACE experimental design. Part of the *caf1* operon is shown, with *caf1A* in green, intergenic region III in orange and *caf1* in cyan. The location of the gene specific primer binding site at the 3′ end of *caf1* is highlighted. Using this primer, cDNA was synthesised and sequenced using the 5’RACE method. Predicted individual transcript sequences are depicted as red lines, where the length of the line represents the length of the sequence read. Only the promoter in the P2 region (responsible for transcription of the polycistronic mRNA) is present, and so the polymerase synthesises cDNA until it dissociates from the DNA. This means the majority of sequence reads continue through intergenic region III into *caf1A*, terminating at different positions. **Figure S5** Sequences obtained by 5′ RACE analysis of *caf1* transcripts. The partial coding sequence of *caf1A*, followed by intergenic region III and the coding sequence of *caf1 *is shown (*caf1* operon, COP) aligned to sequence data obtained from 8 separate 5′ RACE reactions. Regions of similarity are bounded by blue boxes with red text, and regions of complete conservation highlighted in red with white text. The regions corresponding to* caf1A*, intergenic region III and *caf1* are underlined in green, orange and cyan respectively. (PDF 1619 kb)

## Abbreviations

ANOVA: Analysis of variance
CU: Chaperone-usher
EPEC: Enteropathogenic *Escherichia coli*
PCR: Polymerase chain reaction
RACE: Rapid amplification of cDNA ends
RDEC: Rabbit diarrheagenic *Escherichia coli*
RT-PCR: Reverse transcription polymerase chain reaction
T3SS: Type 3 secretion system
