# Supplementary material for: Induction of the immunoprotective coat of Yersinia pestis at body temperature is mediated by the Caf1R transcription factor
Source: BMC Microbiol. 2019 Mar 29;19:68. doi: 10.1186/s12866-019-1444-4 (PMC6440114; doi:10.1186/s12866-019-1444-4)
Supplement: Supplementary file 1 — Table S1 List of constructs used in this study. Table S2 List of primers used in this study. Table S3 Thermal cycling conditions for RT-PCR. Table S4 Thermal cycling conditions for PCR. Table S5 Thermal cycling conditions for 5′ RACE. Figure S1 Schematic of primer design. A diagram of the caf1 operon present in the plasmids used in this study is shown, with arrows corresponding to the forward and reverse primers and placed in the approximate position where they bind. Forward primers are shown on top of the genes and reverse primers shown beneath. (A) Primers used for RT-PCR are shown in red for caf1R, green for caf1M, orange for caf1A and blue for caf1. (B) Primers used for detecting the presence of intergenic regions in cDNA are shown: purple for intergenic region I, olive for intergenic region II and dark blue for intergenic region III. Figure S2 Schematic of the P1, P2 and P3 regions. The sequence of intergenic region I (INT1), located between caf1R and caf1M is shown, highlighted in green, with the P1, P2 and P3 regions aligned and highlighted in red, yellow and orange respectively. The ATG nucleotides corresponding to the start codon of caf1M are labelled with blue triangles. Figure S3 Analysis of Caf1 content in the flocculent layer (A) Image of E. coli cultures containing the pT7-COP plasmid grown at 35 °C for the amounts of time stated, and centrifuged in capillary tubes to visualise the flocculent layer height. (B) SDS-PAGE analysis of the flocculent layers of the cultures from (A). (C) Graph of Caf1 band intensities in arbitrary units, obtained by densitometry of the gel shown in (B). Figure S4 Diagram depicting 5’RACE experimental design. Part of the caf1 operon is shown, with caf1A in green, intergenic region III in orange and caf1 in cyan. The location of the gene specific primer binding site at the 3′ end of caf1 is highlighted. Using this primer, cDNA was synthesised and sequenced using the 5’RACE method. Predicted individual transcript sequences are [file 12866_2019_1444_MOESM1_ESM.pdf]

**Plasmid Name**	**Backbone**	**Genotype**
pCOP	pGEM-T	ΔT7, caf*1R, caf1M, caf1A, caf1*
pCOPΔR	pGEM-T	ΔT7 *Δcaf1R, caf1M, caf1A, caf1*
pT7-COP	pGEM-T	T7, *caf1R, caf1M, caf1A, caf1*
pCOPF	pGEM-T	ΔT7, *caf1R-FLAG, caf1M-FLAG, caf1A-FLAG, caf1*
pCOPFΔR	pGEM-T	ΔT7 *Δcaf1R, caf1M-FLAG, caf1A-FLAG, caf1*
pBad-caf1R	pBad33	*caf1R*

Table S1: List of constructs used in this study.

	Sequence (5’ -3’)	Function
Forward	TCTAGCCGTTCGAATTGGGCCCGACGTC	Substitution of T7 promoter
Reverse	AGAACAAGAACAATTCACTGGCC GTCGTTTTAC	
Forward	TAATCCTAATGTTACAGAATATAACCCAAATCAAAATAATAG	Deletion of Caf1R
Reverse	GTAACATTAGGATTACCAAAGAG	
Forward	TCATCATCATCTTTATAATCACTCTTTGGTAATCCTAATGTTACTGAC	Addition of C-terminal FLAG tag to Caf1R
Reverse	AAAGATGATGATGATAAATAAAATTCCCGCGGCCGCCATG	
Forward	AAAGATGATGATGATAAATAATGATGTTTAAAGGGGACGGG	Addition of C-terminal FLAG tag to Caf1M
Reverse	ATCATCATCATCTTTATAATCT AAAGTCACATTTTTGGAATACAAAC	
Forward	AAAGATGATGATGATAAATAAAACGGATGTTTATTTCAAACAGGACAC	Addition of C-terminal FLAG tag to Caf1A
Reverse	ATCATCATCATCTTTATAATCGTTATTTAAGATGCAGGTTGTGGATAAC	
Forward	TCTGCTGAATCCTGAATACAAAACC	Amplification of intergenic region I
Reverse	CTTGCGAATTTGATATCTGGTTGAG	
Forward	CATGAACATAGGTGAATTAACATTTGG	Amplification of intergenic region II
Reverse	GACGGAAGTCGATATTTCCAGAG	
Forward	AGTATAAGGTAGAAATTAACCCAGTTAC	Amplification of intergenic region III
Reverse	GGAGCGCCTTCCTTATATGTAAG	
Forward	ATGCTAAAACAGATGACTGTAAATTC	RT-PCR, Caf1R
Reverse	AGGCATTCCGACATATTCCTTAAAG	
Forward	ATGATTTTAAATAGATTAAGTACGTTAGG	RT-PCR, Caf1M
Reverse	GCATCTAACGGGTATATGATCC	
Forward	ATGAGGTATTCAAAGCTGTTCCTGTG	RT-PCR, Caf1A
Reverse	TGAAGTCCTTGATTAAAAAGAGATACATCTATAC	
Forward	ATGAAAAAAATCAGTTCCGTTATCGCCATTGC	RT-PCR, Caf1
Reverse	GTAATTGGAGCGCCTTCCTTATATGTAAG	
Forward	ATGAGCACTTTTAAAGTTCTGCTATG	RT-PCR, Ampicillin
Reverse	TGTCATGCCATCCGTAAGATGC	
Gene specific primer	CCATAATTG	5’ RACE
(dT)17-adaptor	GACTCGAGTCGACATCGATTTTTTTTTTTTTTTTT	5’ RACE
Adaptor	GACTCGAGTCGACATCG	5’ RACE

Table S2: List of primers used in this study.

Step	Temperature	Time	Number of Cycles
**Initial Denaturation**	95ºC	10 minutes	1 cycle
**Denaturation**	95ºC	30 seconds	40 cycles
**Annealing**	60ºC	30 seconds	
**Extension**	72ºC	30 seconds	

Table S3: Thermal cycling conditions for RT-PCR

Step	Temperature	Time	Number of Cycles
**Initial Denaturation**	98ºC	3 minutes	1 cycle
**Denaturation**	98ºC	10 seconds	35 cycles
**Annealing**	45ºC - 72ºC	30 seconds	
**Extension**	72ºC	25 seconds	
**Final Extension**	72ºC	10 minutes	1 cycle
**Hold**	4ºC	Indefinite	1 cycle

Table S4: Thermal cycling conditions for PCR

Step	Temperature	Time	Number of Cycles
**Initial Denaturation**	98ºC	3 minutes	1 cycle
**Denaturation**	98ºC	10 seconds	35 cycles
**Annealing**	54 ºC	30 seconds	
**Extension**	72ºC	25 seconds	
**Final Extension**	72ºC	10 minutes	1 cycle
**Hold**	4ºC	Indefinite	1 cycle

Table S5: Thermal cycling conditions for 5’ RACE

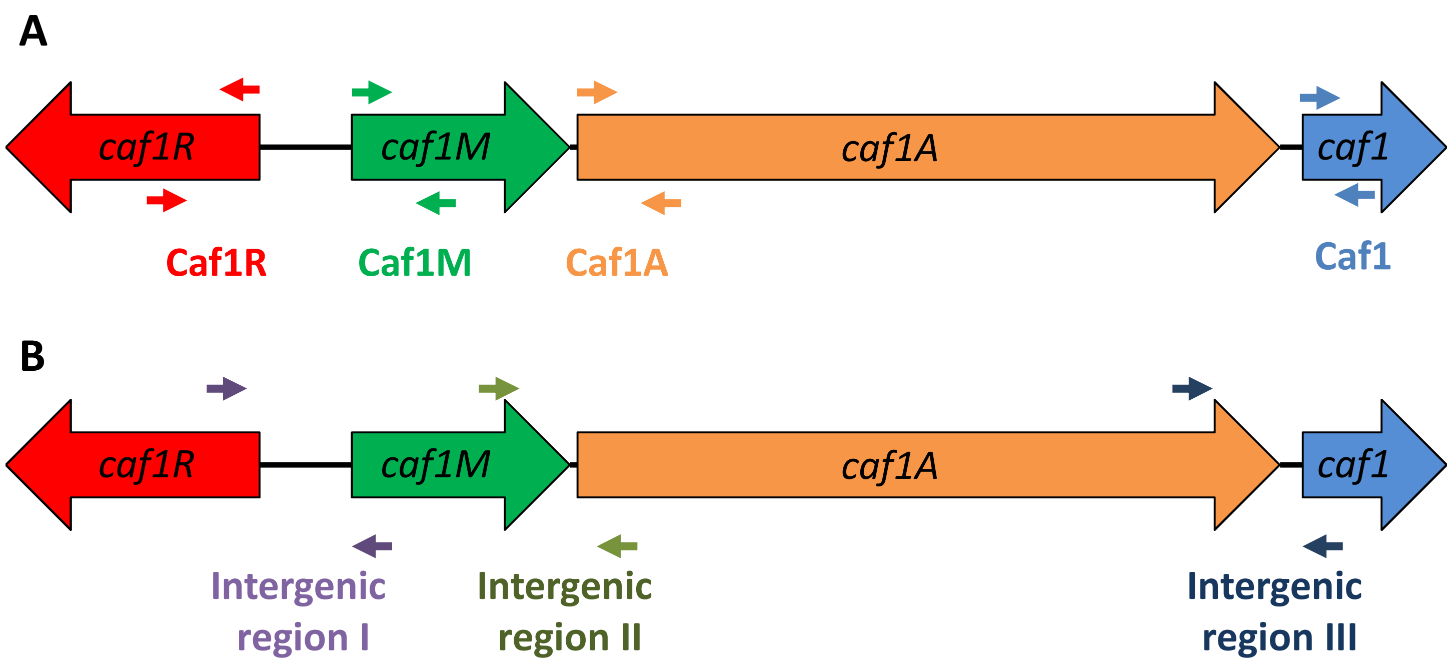

Figure S1: **Schematic of primer design.** A diagram of the *caf1* operon present in the plasmids used in this study is shown, with arrows corresponding to the forward and reverse primers and placed in the approximate position where they bind. Forward primers are shown on top of the genes and reverse primers shown beneath. (A) Primers used for RT-PCR are shown in red for *caf1R*, green for *caf1M*, orange for *caf1A* and blue for *caf1*. (B) Primers used for detecting the presence of intergenic regions in cDNA are shown: purple for intergenic region I, olive for intergenic region II and dark blue for intergenic region III.

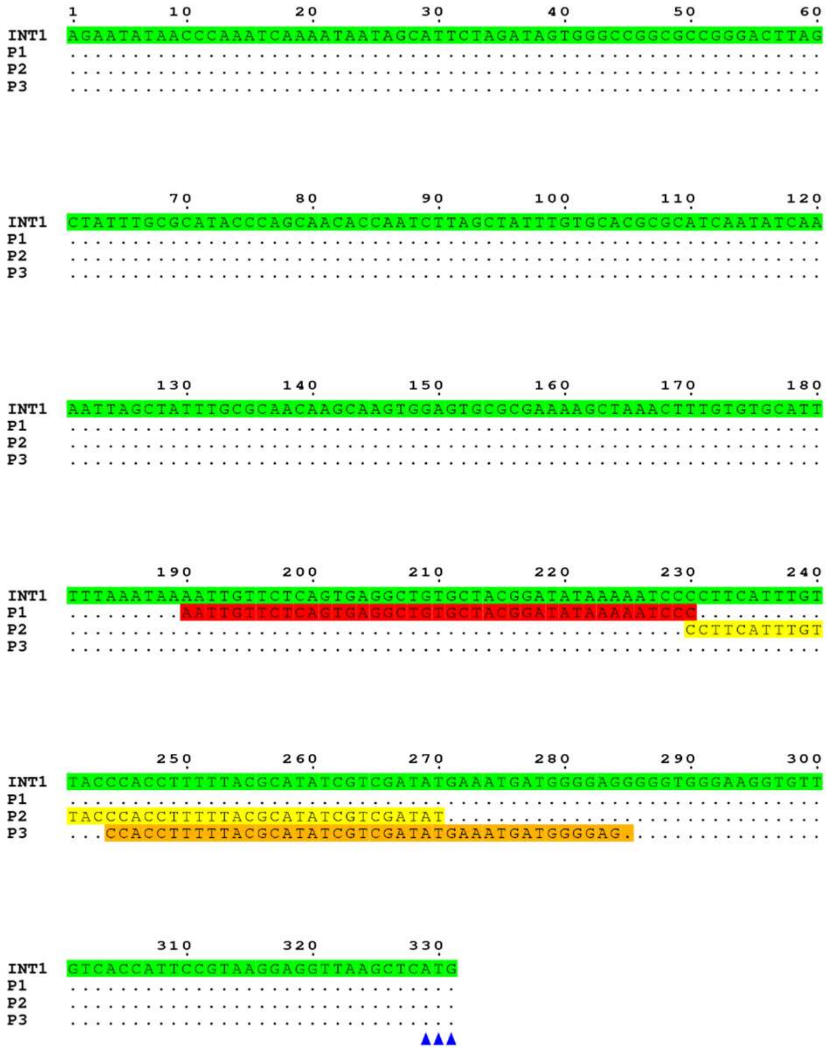

Figure S2: **Schematic of the P1, P2 and P3 regions.** The sequence of intergenic region I (INT1), located between *caf1R* and *caf1M* is shown, highlighted in green, with the P1, P2 and P3 regions aligned and highlighted in red, yellow and orange respectively. The ATG nucleotides corresponding to the start codon of *caf1M* are labelled with blue triangles.

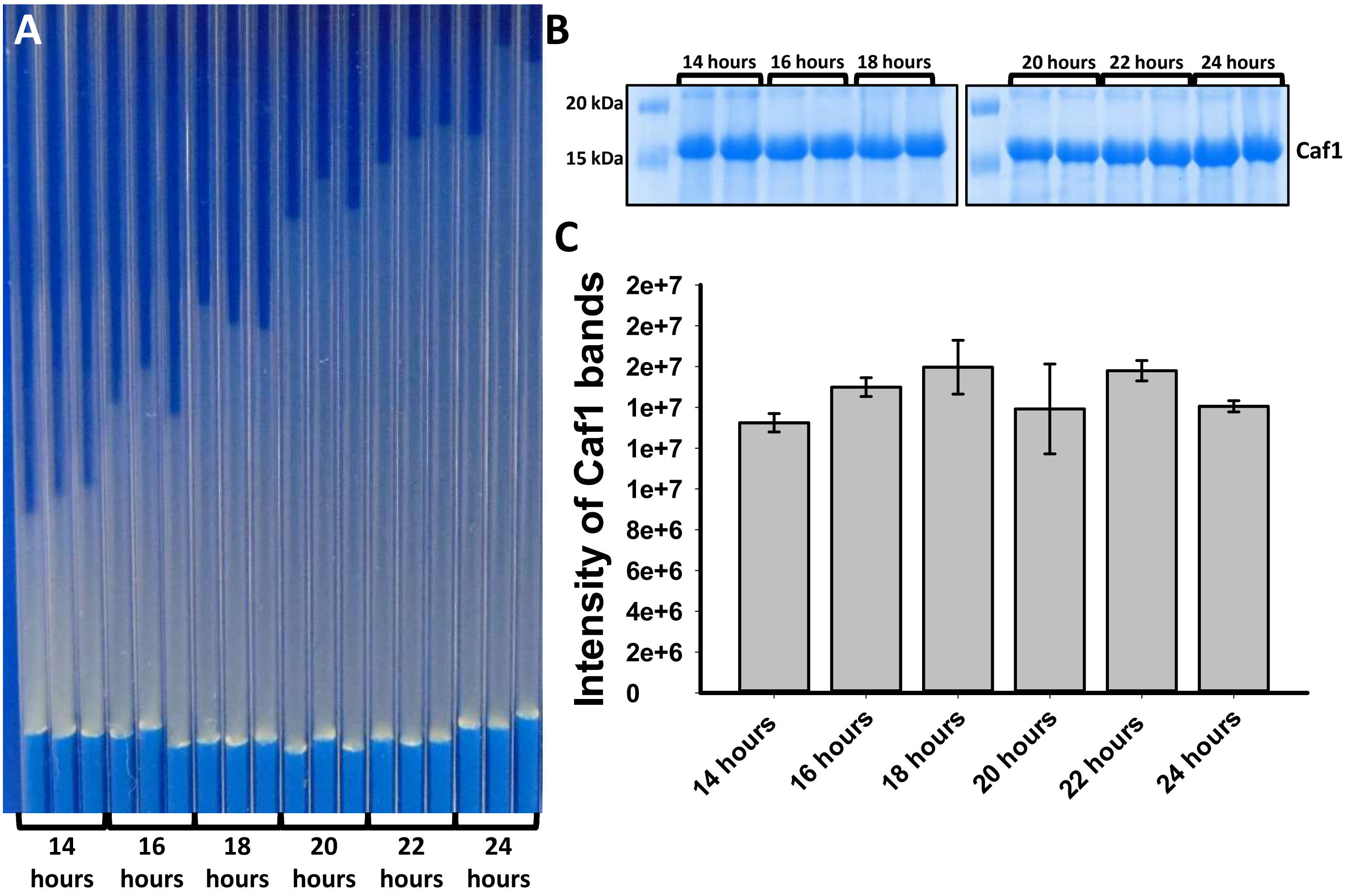

Figure S3: **Analysis of Caf1 content in the flocculent layer** (**A**) Image of *E. coli* cultures containing the pT7-COP plasmid grown at 35°C for the amounts of time stated, and centrifuged in capillary tubes to visualise the flocculent layer height. (**B**) SDS-PAGE analysis of the flocculent layers of the cultures from (**A**). (**C**) Graph of Caf1 band intensities in arbitrary units, obtained by densitometry of the gel shown in (**B**).

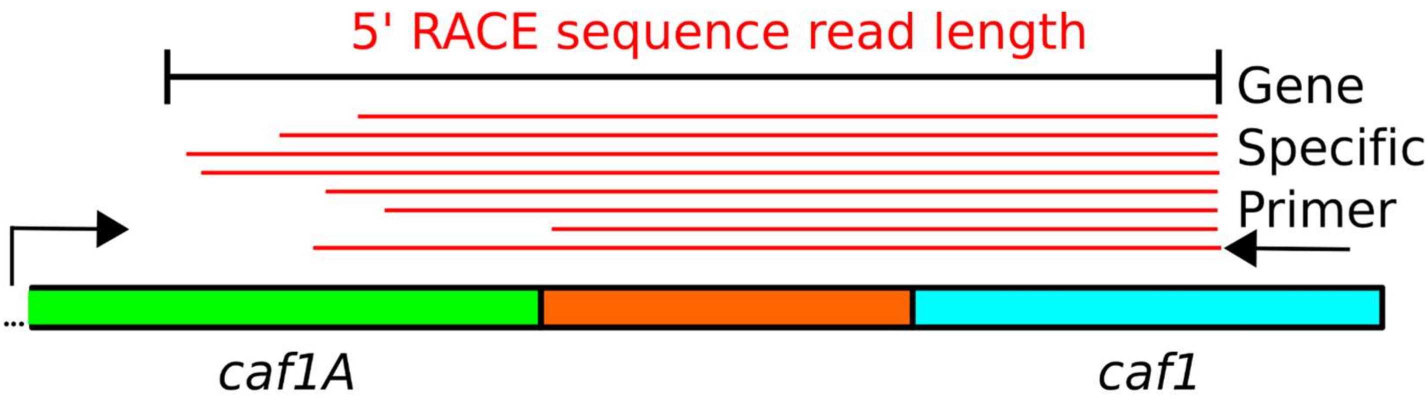

Figure S4: Diagram depicting 5'RACE experimental design. Part of the *caf1* operon is shown, with *caf1A* in green, intergenic region III in orange and *caf1* in cyan. The location of the gene specific primer binding site at the 3' end of *caf1* is highlighted. Using this primer, cDNA was synthesised and sequenced using the 5'RACE method. Predicted individual transcript sequences are depicted as red lines, where the length of the line represents the length of the sequence read. Only the promoter in the P2 region (responsible for transcription of the polycistronic mRNA) is present, and so the polymerase synthesises cDNA until it dissociates from the DNA. This means the majority of sequence reads continue through intergenic region III into *caf1A*, terminating at different positions.

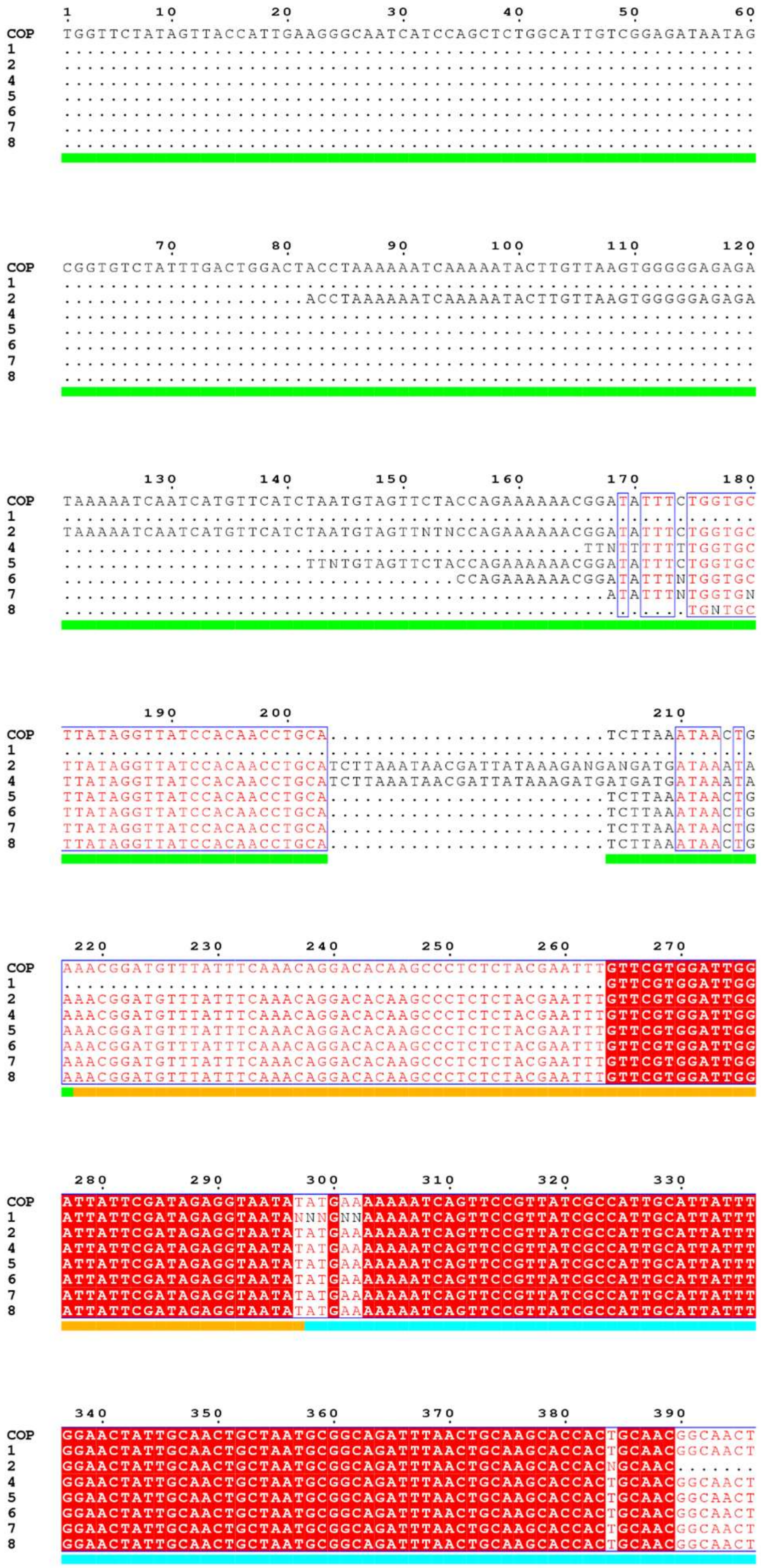

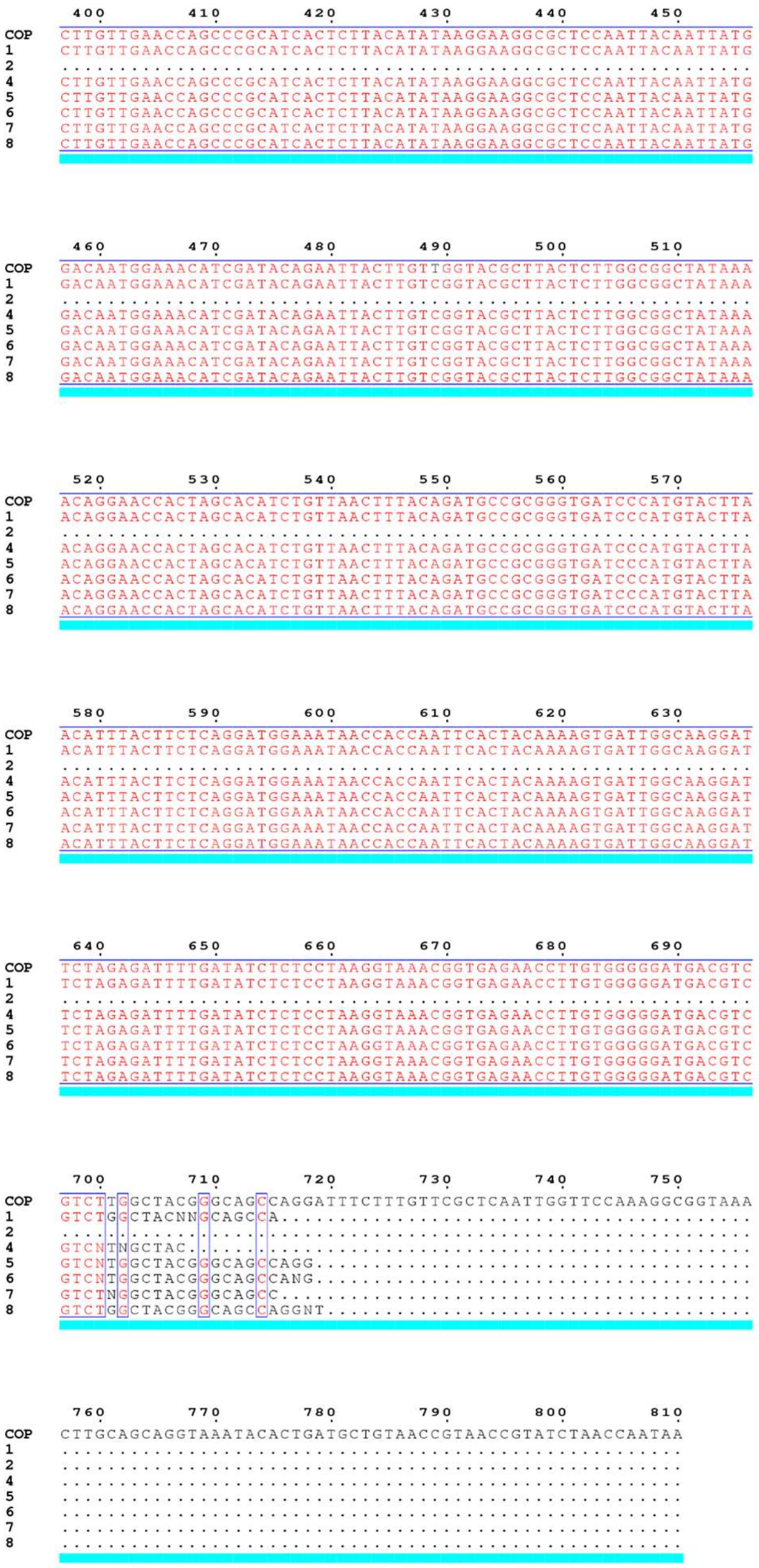

Figure S5: Sequences obtained by 5' RACE analysis of *caf1* transcripts. The partial coding sequence of *caf1A*, followed by intergenic region III and the coding sequence of *caf1* is shown (***caf1* op**eron, COP) aligned to sequence data obtained from 8 separate 5' RACE reactions. Regions of similarity are bounded by blue boxes with red text, and regions of complete conservation highlighted in red with white text. The regions corresponding to *caf1A*, intergenic region III and *caf1* are underlined in green, orange and cyan respectively.
